# Rumen Microbial Metabolic Responses of Dairy Cows to the Honeycomb Flavonoids Supplement Under Heat-Stress Conditions

**DOI:** 10.3389/fvets.2022.845911

**Published:** 2022-03-15

**Authors:** En Liu, Mengxue Sun, Chenxin He, Kang Mao, Qin Li, Jianhong Zhang, Deyong Wu, Shuzhen Wang, Chuanxia Zheng, Wenbin Li, Shimin Gong, Fuguang Xue, Huadong Wu

**Affiliations:** ^1^Nanchang Key Laboratory of Animal Health and Safety Production, Jiangxi Agricultural University, Nanchang, China; ^2^Jiangxi Province Key Laboratory of Animal Nutrition/Engineering Research Center of Feed Development, Jiangxi Agricultural University, Nanchang, China

**Keywords:** honeycomb flavonoids, dairy cows, heat stress, metagenomics, metabolomics

## Abstract

Flavonoids played critical roles in stabilizing microbial homoeostasis when animals suffered exoteric stresses. However, whether flavonoids attenuated heat stress of dairy cows is still not clear. Therefore, in the present article, flavonoids extracted from honeycomb were supplemented to investigate the production, digestibility, and rumen microbial metabolism responses of cows under heat stress conditions. A total of 600 multiparous dairy herds were randomly allotted into the control treatment (CON), the heat stress (HS) treatment, and the honeycomb flavonoids supplement under heat stress conditions (HF) treatment for a 30-day-long trial. Each treatment contains 4 replicates, with 50 cows in each replicate. Production performances including dry matter intake (DMI), milk production, and milk quality were measured on the basis of replicate. Furthermore, two cows of each replicate were selected for the measurement of the nutrient digestibility, the ruminal fermentable parameters including ruminal pH, volatile fatty acids, and ammonia-N, and the rumen microbial communities and metabolism. Results showed that HF effectively increased DMI, milk yield, milk fat, and ruminal acetate content (*p* < 0.05) compared with HS. Likewise, digestibility of NDF was promoted after HF supplement compared with HS. Furthermore, relative abundances of rumen microbial diversities especially *Succiniclasticum, Pseudobutyrivibrio, Acetitomaculum, Streptococcus*, and *Succinivibrio*, which mainly participated in energy metabolism, significantly improved after HF supplement. Metabolomic investigation showed that HF supplement significantly upregulated relative content of lipometabolic-related metabolites such as phosphatidylglycerol, phosphatidylinositol, phosphatidylserine, and phosphatidylethanolamine, while it downregulated biogenic amines. In summary, HF supplement helps proliferate microbial abundances, which further promoted fiber digestibility and energy provision, and ultimately enhances the production performances of dairy cows under heat stress conditions.

## Introduction

Heat stress traditionally refers to the combination of the excessive heat accumulation and the reductive heat dissipation, which may seriously induce fervescence (1, 2), the reduction of feed intake (3), the turbulence of rumen microbiota, and the reduction of milk production of dairy cows (4). During the last few decades, researchers dedicated to find the proper methods in ameliorating heat stress during dairy cow production and proposed certain solutions including spraying, fans, shading, and nutritional methods (3).

The most commonly applied nutritional methods to ameliorate heat stress is to enhance rumen digestibility of dairy cows through the addition of minerals, vitamins, prebiotics, and probiotics with balanced nutrition (5). Compared with the initial supplement of nutritional elements, microbial secondary metabolites, such as flavonoids, played an essential role in shaping microbial communities, maintaining homoeostasis, and promoting host growth (6) by their splendid antioxidation and free radical scavenging capabilities (7–9). Moreover, flavonoids also regulated nutrient absorptivity by enhancing ruminal epithelial development (10). Li et al. (11) proved the ameliorative effects of flavonoids on dairy cows under heat stress conditions. In contrast, how flavonoids worked on ruminal microbial communities and metabolism is still not clear.

Rumen microbiota is the stabilized and powerful self-regulated digestive ecosystem that converts feed nutrients into absorbable compounds for the host (12). Ruminal microbiota communities also showed the accommodated responses to heat stress, such as the decrease of higher fiber degradative bacteria, *Fibrobacter*, and the increase of starch-utilizing bacteria, *Clostridium* and *Streptococcus*, which further reduced heat generation and utilized more easily fermented carbohydrates to maintain the energy provision (13). Microbial metabolites are subsequently altered to adapt to the change of microbial communities, and further regulated the growth and production of the host (14). Disorders of ruminal microbial ecosystem and the following declines of nutritional digestibility when subjected to heat stress mainly caused the reduction of dairy production (15, 16). Therefore, the most critical point in attenuating heat stress is the re-establishment of the physiological thermal energy balance between heat acquisition and heat dissipation through the regulation of ruminal microbial community and nutrient metabolism.

In the present article, flavonoids extracted from honeycomb were supplemented to investigate the productive, rumen microbial, and rumen metabolism responses of cows under heat stress conditions, which aimed to provide a selectable suggestion for dairy production. We hypothesized that flavonoids help promote the proliferation of fiber-degrading bacteria, following the regulation of energy metabolism, and ultimately enhanced the production performances of dairy cows.

## Materials and Methods

Animal care and procedures followed the Chinese Guidelines for Animal Welfare, which was approved by the Animal Care and Use Committee of Jiangxi Agricultural University, with the approval number JXAULL-20210619.

### Experimental Design

The experiment was conducted in the Bengbu dairy farm, Modern Farming (Wuhe) Co. Ltd., Anhui Province, China (32.92N, 117.38E) from July 7, 2021 to August 7, 2021. A total of 600 multiparous dairy herds with an average live weight of 643.6 ± 37.3 kg, average lactation of 159.3 ± 18.8 days, and average calving parities of 2.58 ± 0.49 were randomly allocated into the control treatment (CON), the heat stress treatment (HS), and honeycomb flavonoids supplement under heat stress conditions treatment (HF). Each treatment was distributed to an individual stall, and with the use of a transverse ventilation system, the wind intensity could be regulated by automatic ventilators. Each stall contains four pens and 50 cows in each pen. Each pen was considered as a replicate and arranged along the wind direction to ensure that each pen receives similar wind intensity. During the trial period, cows in control treatment received the spraying treatment to ensure non-occurrence of heat stress. No cooling methods were provided for the HS and HF treatments.

All cows were provided the same diets, which were formulated according to NRC (2001) to meet or exceed the energy requirements of Holstein dairy cows yielding 30 kg of milk/day with 3.5% milk fat and 3.0% true protein. Diets were fed three times per day at 06:00, 13:00, and 21:00. Details of ingredient analysis and chemical composition of diets are shown in Table 1.

**Table 1:** Ingredients and chemical composition of the TMR (dry-matter basis).

**Items**	**Content**
**Ingredients (%)**	
Corn silage	24.5
Corn	15.7
Cottonseed meal	3.4
Alfalfa hay	14.5
Chinese wild rye	10.2
Distillers' dried grains with solubles (DDGS)	3.1
Steam-flaked corn	8.5
Soybean meal	12.0
Beet pulp	4.5
Premix^a^	3.0
NaCl	0.6
Total	100
**Chemical composition (%)**	
NE (MJ/kg)	7.13
EE	4.56
CP	17.1
ADF	18.6
NDF	31.7
Ca	0.69
P	0.44

a *The components contained in the premix are as follows: Fe, 1,400 mg; Cu, 1,200 mg; Mn, 2,400 mg; Zn, 5,500 mg; Se, 40 mg; Co, 30 mg; I, 90 mg; VA, 900,000 IU; VD, 700,000 IU; VE, 9,000 IU*.

Temperature and humidity of each feeding lair were recorded every day, and the temperature humidity indexes (THI) were calculated through the following equation:


$$\begin{array}{l} \text{THI}=\left(1.8\times \text{T}+32\right)-\left[\left(0.55-0.0055\times \text{RH}\right)\right]\times \\ \;\left(1.8\times \text{T}-26\right) \end{array}$$

where T = temperature (°C), RH = relative humidity (%).

THI ≥70 was considered as heat stress occurring in high-yielding dairy cows (17). Body temperature was measured through rectal thermometry once a week through a 30-size sampling inspection of each treatment.

Raw honeycomb was provided by the Institute of Bee Research, Jiangxi Agricultural University, Jiangxi, China. HF was further extracted in the laboratory using an ultrasonic (Elmasonic X-tra Flexl; Elma, Konstanz, Germany) extracting procedure combined with the solid-to-liquid ratio of 1:29.76, 56.33% of ethanol concentration, and the reaction time of 28.88 min. Extractions were further centrifugated at 3,000 rpm for 10 min. The postextracting liquor was filtered after cooling and collected into an Erlenmeyer flask.

Flavonoids content measurement were subsequently applied based on the Chinese National standard GB/T 20574-2006. To be simply stated, flavonoids standard samples were first precisely weighed at 10 mg and dissolved into 50 ml ethanol. Gradients of 1, 2, 3, 4, 5, and 6 ml of the standard solution were collected into a 50-ml volumetric flask, and isochorically added to 15 ml of 95% ethanol. Then, 1 ml of 100 g/L aluminum nitrate solution and 1 ml of 9.8 g/L potassium acetate solution were added into the mixture, respectively. Absorption of each standard solution was measured at 415 nm wavelength after standing for 60 min. The standard curve was then made as follows:


$$\begin{array}{l} \text{y}=0.4145\text{x}+0.001\left({\text{R}}^{2}=\;0.9997\right) \end{array}$$

where y = flavonoids content and x = absorption.

Honeycomb flavonoids content was measured about 45% of the standard flavonoids and supplemented with 10 g/day for each cow.

### Production Performance Measurement

Average daily intake was shown as the dry matter intake (DMI) of each treatment, which were calculated through the deviation between the supply and residue. DMI were calculated and displayed as the average of four replicates. Cows were milked three times per day (08:00, 14:00, and 20:00, respectively) and the daily milk production was automatically recorded through the rotary milking facilities (9JRP-50P2100; Delaval, Israel). Milk samples were collected in 100-ml vials from each replicate at the last day and subsequently stored at 4°C with the addition of 2-bromo-2-nitropropan-1,3-diol for further milk quality analysis including milk protein, milk fat, and somatic cell count.

### Ruminal Fermentation Parameter Measurement

Rumen fluid samples of 24 cows (two cows in each replicate, eight cows per treatment, second lactation, similar body weight) were collected at the last day through esophageal tubing at 3 h after morning feeding. All samples were divided into two portions. One was conducted to analyze ruminal pH, rumen volatile fatty acids (VFAs), and ammonia-N (NH_3_-N). The other portion was frozen in the liquid nitrogen immediately and then stored at −80°C for further microbiota and metabolite measurement. Portable type pH meter (Testo 205; Testo AG, Lenzkirch, Germany) was applied for the measurement of ruminal pH immediately after rumen fluid sample was collected. Individual and total VFAs in the aliquots were measured using a gas chromatograph (GC-2010; Shimadzu, Kyoto, Japan). Concentration of NH_3_-N was determined by indophenol method and the absorbance value was measured through UV-2600 ultraviolet spectrophotometer (Tianmei Ltd., China) at the 700 nm wavelength (12).

### Digestibility Measurement

Digestibility measurement was conducted during the last 3 days. To be simply stated, total fecal samples were collected at intervals of 6 h (18 samples in total) after 12-h-long fasting treatment. Digestibility of dry matter (DM), crude protein (CP), neutral detergent fiber (NDF), and fatty acids (EE) were subsequently measured based on the methods we stated previously (18).

### Rumen Microbial Community Measurement

Rumen fluid DNA was extracted using Bacterial Genome DNA Extraction Kit (DP302, TIANGEN; TIANGEN BIOTECH (BEIJING) Co., Ltd). The V4 region of the 16S rRNA gene was amplified using the universal primers 520F and 802R (F: GTGCCAGCMGCCGCGGTAA and R: GGACTACHVGGGTWTCTAAT) (18). All PCR reactions were carried out with Phusion High-Fidelity PCR Master Mix (New England Biolabs). The mixture of PCR products was purified with Qiagen Gel Extraction Kit (Qiagen, Hilden, Germany), followed by the generation of sequencing libraries using TruSeq DNA PCR-Free Sample Preparation Kit (Illumina, USA). The library quality was assessed on the Qubit@ 2.0 Fluorometer (Thermo Scientific) and Agilent Bioanalyzer 2100 system. Finally, the library was sequenced on Illumina HiSeq 4000 platform (Illumina Inc., San Diego, USA).

Quality filtering of raw tags were performed under specific filtering conditions to obtain the high-quality clean tags according to the Quantitative Insights Into Microbial Ecology (QIIME, V1.7.0) quality controlling process. Sequences with >97% similarity were assigned into the same operational taxonomic unit (OTU).

### Rumen Metabolite Measurement

Rumen metabolomics in the present study were measured using GC/MS analysis method through Agilent 7890 gas chromatograph system coupled with a Pegasus HT time-of-flight mass spectrometer (LECO, St. Joseph, MI). The main process was detailed as stated in our previous study (19). To simply state, in all samples were added 55 μl of methoxy amination reagent (20 mg/ml dissolved in pyridine) followed by a 20-min-long incubation at 80°C. Subsequently, all samples were incubated for 1 h at 70–80°C after adding 75 μl BSTFA reagent (1% TMCS, v/v). Thereafter, 10 μl fatty acid methyl ester (FAMEs) (standard mixture of fatty acid methyl esters, C8–C16:1 mg/ml; C18–C24:0.5 mg/ml in chloroform) was added into each sample after all samples were cooled to room temperature. Finally, all samples were prepared for GC–MS analysis after mixing them well.

### Statistical Analysis

Body temperature; production performances including DMI and milk yield; milk quality parameters including milk fat, milk protein, and SCC; and ruminal fermentation variables including ruminal pH, VFAs, and ammonia-N were first determined by conducting a normal distribution test using SAS procedure “proc univariate data=test normal” and subsequently received one-way ANOVA S-N-K test by SAS (SAS Institute, Inc., Cary, NC, USA). Significance would be considered when *p*-value <0.05 while a tendency was considered when 0.05 ≤ *p* < 0.10. OTU abundances of each rumen bacteria were first determined by conducting a percentage transformation, and then one-way ANOVA S-N-K test of SAS 9.2 was applied for the differential analysis. Alpha diversity and beta diversity in our samples were calculated with QIIME 2 (20) and displayed with R software (version 3.3.1; R Core Team, Vienna, Austria). Principal coordinate analysis (PCoA) for different rumen methanogens were conducted using R “vegan package”.

For metabolomics data analysis, Chroma TOF 4.3X software of LECO Corporation and LECO-Fiehn Rtx5 database were used for peak identification and integration of the peak area. Multivariate analysis including principal component analysis (PCA) were conducted using SIMCA-P software (V.14.0; Umetrics, Umea, Sweden). Differentially expressed metabolites between two treatments were identified based on variable importance in projection (VIP) and statistical analysis (VIP > 1 and *p* < 0.05). Functional enrichment analysis was conducted using Metaboanalyst 5.0 (https://www.metaboanalyst.ca/). Significant function enrichment would be considered when *p*-value <0.05.

## Results

### THI Recording

**Figure 1:**
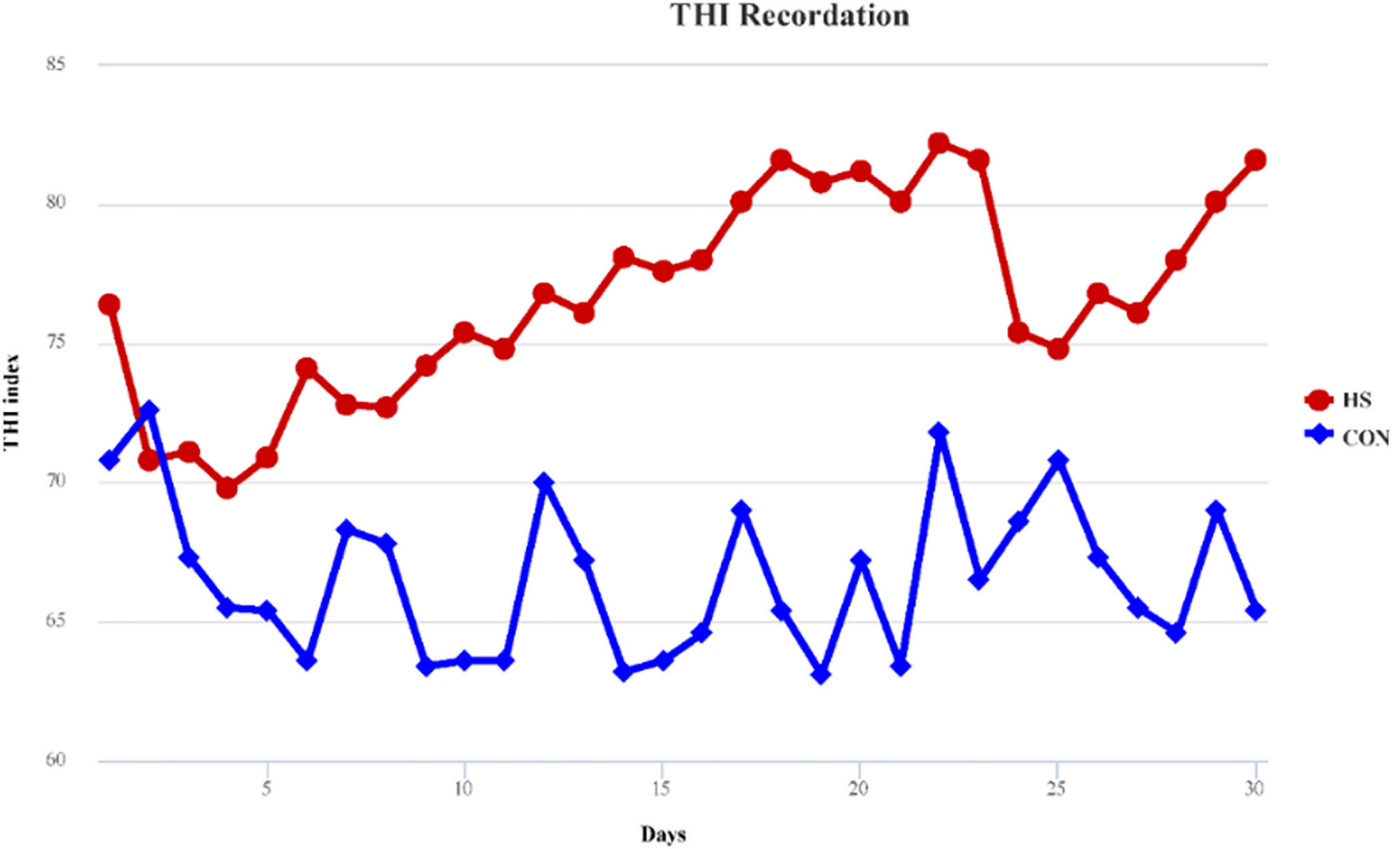
THI record of both heat stress and control treatments. HS, heat stress; CON, control treatment.

In our study, temperature and relative humidity of each treatment were recorded in five different locations, and the THI was calculated based on the average temperature and humidity. THI was recorded throughout the whole experimental period and displayed in Figure 1. During the entire experimental period, environmental THI exceeded 70 for HS treatments, while during 27 days of CON treatment, it was below 70. HF treatment showed the same THI with HS for the cows that received HF treatment and reared in the same stall with HS. THI provided a confirmation of the occurrence of heat stress.

### Production Performances and Milk Quality

The production parameters for dairy cows were first recorded, and the results are shown in Table 2. Heat stress significantly restrained DMI and milk yield compared with CON treatment, while both parameters increased after honeycomb flavonoids supplement, however, not significantly. Besides, honeycomb flavonoids significantly reduced the milk somatic cell counts, which might be the indicator of mastitis, and remarkably increased under heat stress conditions. Body temperature in HS treatment significantly increased compared with CON, while it decreased after honeycomb flavonoids supplement. There is an increasing trend of milk fat after HF treatment, while no significant changes were found for milk protein content.

**Table 2:** Effects of honeycomb flavonoids supplement on production performances and milk quality during heat stress conditions.

**Items**	**Experimental treatments**			**SEM**	***P*-value**
	**CON (*n* = 4)**	**HS (*n* = 4)**	**HF (*n* = 4)**		
Body temperature (°C)	38.7^b^	39.1^a^	38.8^b^	0.10	0.046
DMI (kg)	22.9^a^	21.4^b^	22.3^ab^	0.68	0.033
Milk yield (kg)	31.6^a^	29.4^b^	30.3^ab^	0.76	0.036
Milk fat (%)	3.67	3.63	3.68	0.06	0.056
Milk protein (%)	3.38	3.34	3.37	0.03	0.412
SCC, ×10^4^ cells/ml	12.64^b^	19.39^a^	13.77^b^	2.46	0.033

a,b *Means within a row with different letters differed significantly (p < 0.05). CON, control treatment; HS, heat stress treatment; HF, honeycomb flavonoids treatment; DMI, dry matter intake; SCC, somatic cell counts*.

### Rumen Fermentability and Nutrient Digestibility

Rumen fermentability, including ruminal pH, VFAs, and ammonia-N, were measured, and the results are shown in Table 3. Heat stress reduced ruminal acetate, propionate, and butyrate content while the acetate and ratio of acetate/propionate significantly decreased (*p* < 0.05). Acetate content increased significantly after HF supplement (*p* < 0.05), although still inferior to CON treatment. In addition, the ratio of acetate/propionate in HF were significantly higher than that in HS. No significant differences were detected for other parameters among CON, HS, and HF treatments.

**Table 3:** Effects of honeycomb flavonoids supplement on rumen fermentable parameters during heat stress conditions.

**Items**	**Experimental treatments**			**SEM**	***P*-value**
	**CON (*n* = 8)**	**HS (*n* = 8)**	**HF (*n* = 8)**		
Ruminal pH	6.12^b^	6.23^a^	6.22^a^	0.094	0.016
Acetate (mmol/L)	49.24^a^	43.62^c^	46.07^b^	1.273	0.038
Propionate (mmol/L)	14.49	13.75	13.44	0.632	0.076
Acetate/propionate	3.40^a^	3.17^b^	3.43^a^	0.023	0.043
Butyrate (mmol/L)	9.77	9.35	9.82	0.137	0.356
Isobutyrate (mmol/L)	2.23	2.08	2.11	0.115	0.642
Valerate (mmol/L)	2.13	2.21	2.14	0.064	0.744
Isovalerate (mmol/L)	2.31	2.26	2.46	0.16	0.915
Ammonia-N (mg/100 ml)	12.49	11.96	12.27	1.711	0.622

a,b,c *Means within a row with different letters differed significantly (p < 0.05). CON, control treatment; HS, heat stress treatment; HF, honeycomb flavonoids treatment*.

Furthermore, nutrient digestibility was measured through total feces collection method, and the results are shown in Table 4. Digestion of dry matter, NDF, and fatty acids was enhanced after HF supplement while the digestibility of NDF increased significantly compared with HS. No significant changes were found for the CP digestion.

**Table 4:** Effects of honeycomb flavonoids supplement on cow digestibility under heat stress conditions.

**Items**	**CON**	**HS**	**HF**	**SEM**	***P*-value**
DM	64.03	62.86	63.83	0.71	0.176
NDF	68.64^a^	66.08^b^	68.62^a^	0.32	0.001
CP	62.14	62.48	61.87	0.49	0.595
EE	74.75	73.96	75.37	1.18	0.181

a,b *Means within a row with different letters differed significantly (p < 0.05). CON, control treatment; HS, heat stress treatment; HF, honeycomb flavonoids supplement under heat stress conditions; DM, dry matter; NDF, neutral detergent fiber; CP, crude protein; EE, ether extracts*.

### Rumen and Microbial Communities

A total of 4,700 OTUs, 15 phyla, and 2,600 genera were identified after quality control, and all the taxonomic information is displayed in Supplementary Table 1. All identified bacteria were chosen for further investigation of α-diversity, β-diversity, and differential communities.

#### α-Diversity

Alpha diversity was first investigated to determine the complexity of rumen microbial diversity through Chao1, Shannon, Simpson, and ACE indexes; all results are shown in Table 5. Alpha-diversity indexes including ACE and Chao1 significantly decreased under heat stress conditions (*p* < 0.05). Meanwhile, the aforementioned indexes increased after HF supplement, however, not significantly. No changes were detected for other indexes among all the three treatments.

**Table 5:** Effects of honeycomb flavonoids supplement on α-diversity of ruminal microbiota during heat stress conditions.

**Items**	**Experimental treatments**			**SEM**	***P*-value**
	**CON (*n* = 8)**	**HS (*n* = 8)**	**HF (*n* = 8)**		
Shannon	7.26	7.11	7.16	0.15	0.182
Simpson	0.98	0.98	0.98	0.01	0.342
ACE	2,356.5^a^	2,171.4^b^	2,203.4^b^	52.4	0.018
Chao1	2,256.3^a^	2,116.6^b^	2,131.4^b^	51.2	0.062

a,b *Means within a row with different letters differed significantly (p < 0.05). CON, control treatment; HS, heat stress treatment; HF, honeycomb flavonoids treatment*.

#### β-Diversity

**Figure 2:**
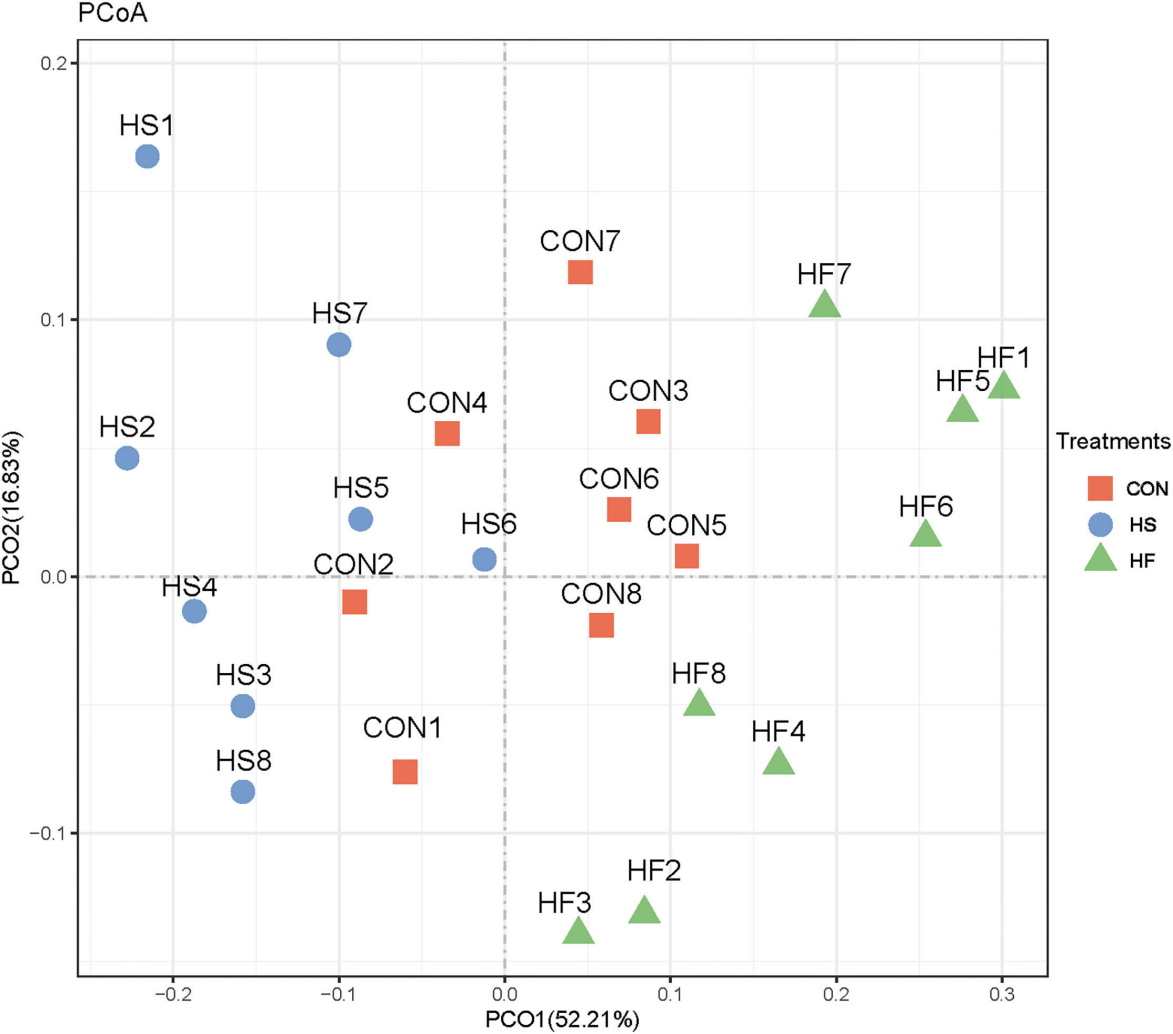
Principal coordinate analysis (PCoA) on ruminal bacterial community structures of CON, HS, and HF treatments. CON, control treatment; HS, heat stress treatment; HF, honeycomb flavonoids supplement treatment.

Subsequently, differential analyses on rumen microbial communities among the three treatments were applied. Principal coordinates analysis (PCoA) analysis was first proceeded. As shown in Figure 2, PCoA axes 1 and 2 accounted for 52.21 and 16.83%, respectively. Bacterial communities in HS treatment could be significantly separated from those in CON through PCoA axes 1 and 2, except HS6. Meanwhile, bacterial communities were significantly altered after HF supplement compared with heat stress conditions.

Thereafter, differential analyses on relative abundances of ruminal bacterial communities in the level of phyla and genera were determined. As the results have shown in Table 6, *Firmicutes* and *Bacteroidetes* contributed the top 2 bacterial biomass. Heat stress treatment significantly proliferated the relative abundance of *Firmicutes*, while it suppressed that of *Bacteroidetes*. Honeycomb flavonoids slightly increased the relative abundances of *Bacteroidetes* while it reduced *Firmicutes*, however, not significantly. It was especially noticed that HF supplement significantly increases the abundance of *Proteobacteria*. No changes were found for other phyla among all the three treatments.

**Table 6:** Effects of honeycomb flavonoids supplement on relative abundances of ruminal microbiota communities during heat stress conditions (level of phyla, %).

**Items**	**Experimental treatments**			**SEM**	***P*-value**
	**CON (*n* = 8)**	**HS (*n* = 8)**	**HF (*n* = 8)**		
*p__Actinobacteria*	0.15	0.25	0.21	0.07	0.229
*p__Fibrobacteres*	0.019	0.013	0.017	0.004	0.186
*p__Bacteroidetes*	67.78^a^	62.78^b^	64.76^b^	2.16	0.028
*p__Firmicutes*	29.92^b^	34.35^a^	32.34^a^	1.23	0.041
*p__Tenericutes*	1.22	1.41	1.35	0.21	0.101
*p__Cyanobacteria*	0.01	0.02	0.01	0.01	0.335
*p__Patescibacteria*	0.10	0.13	0.18	0.05	0.266
*p__Proteobacteria*	0.17^b^	0.17^b^	0.36^a^	0.06	0.004
*p__Spirochaetes*	0.53	0.72	0.68	0.11	0.133
Others	0.12	0.16	0.17	0.04	0.122

a,b *Means within a row with different letters differed significantly (p < 0.05)*.

*CON, control treatment; HS, heat stress treatment; HF, honeycomb flavonoids treatment*.

As referred to the genera level, shown in Table 7, *Prevotella, Ruminococcaceae, Succiniclasticum, Lachnospiraceae*, and *Eubacterium* located the five most abundant genera and accounted nearly 90% of all microbiota profiles. Microbial communities including *Succiniclasticum, Eubacterium, Acetitomaculum, Bifidobacterium*, and *Succinivibrio* significantly proliferated while the growth of *Ruminococcaceae, Ruminococcus, Pseudobutyrivibrio*, and *Streptococcus* significantly suppressed under heat stress conditions. However, HF supplement effectively attenuated these changes mainly manifested in the increasing abundance of *Succiniclasticum, Acetitomaculum, Bifidobacterium*, and *Succinivibrio*, and the decrease of *Ruminococcaceae* and *Ruminococcus*. Noticeably, the main starch-degrading bacteria such as *Pseudobutyrivibrio* and *Streptococcus* increased after HF supplement compared with HS treatment.

**Table 7:** Effects of honeycomb flavonoids supplement on relative abundances of ruminal microbiota communities during heat stress conditions (level of genera, %).

**Items**	**CON (*n* = 8)**	**HS (*n* = 8)**	**HF (*n* = 8)**	**SEM**	***P*-value**
*g__Prevotella*	20.32	16.75	18.66	1.239	0.064
*g__Ruminococcaceae*	15.84^b^	20.20^a^	15.64^b^	0.189	0.005
*g__Succiniclasticum*	10.89^a^	9.76^b^	11.42^a^	0.321	0.029
*g__Lachnospiraceae*	5.22	5.41	5.38	0.282	0.623
*g__Eubacterium*	4.84^a^	4.07^b^	4.49^ab^	0.206	0.041
*g__Ruminococcus*	3.77^b^	4.95^a^	4.37^ab^	0.256	0.015
*g__Shuttleworthia*	2.72	1.37	1.95	0.182	0.272
*g__Prevotellaceae*	1.27	1.25	1.28	0.237	0.371
*g__Acetitomaculum*	1.32^E+00^	7.35^E−01^	9.94^E−01^	0.321	0.063
*g__Lachnoclostridium*	6.58^E−01^	7.51^E−01^	7.10^E−01^	0.012	0.137
*g__Butyrivibrio*	4.43^E−01^	4.05^E−01^	4.28^E−01^	0.134	0.395
*g__Ruminiclostridium*	2.07^E−01^	2.84^E−01^	2.45^E−01^	0.041	0.108
*g__Pseudobutyrivibrio*	1.16^E−01^^b^	1.82^E−01^^a^	1.47^E−01^^ab^	0.014	0.009
*g__Selenomonas*	1.08^E−01^	4.00^E−02^	6.66^E−02^	0.021	0.167
*g__Lactobacillus*	5.35^E−02^	6.23^E−02^	5.84^E−02^	0.010	0.089
*g__Bifidobacterium*	2.79^E−02^	2.16^E−02^	2.48^E−02^	0.013	0.048
*g__Escherichia-Shigella*	1.50^E−02^	1.47^E−02^	1.51^E−02^	0.008	0.571
*g__Bacteroides*	1.32^E−02^	1.77^E−02^	1.54^E−02^	0.006	0.345
*g__Succinivibrio*	2.57^E−02^^a^	7.02^E−03^^c^	1.36^E−02^^b^	0.002	0.033
*g__Streptococcus*	7.85^E−03^^b^	1.39^E−02^^a^	1.06^E−02^^ab^	0.003	0.029
*g__Butyricicoccus*	5.36^E−03^	6.20^E−03^	5.83^E−03^	0.006	0.132

a,b,c *Means within a row with different letters differed significantly (p < 0.05)*.

*CON, control treatment; HS, heat stress treatment; HF, honeycomb flavonoids treatment*.

### Effects of Honeycomb Flavonoids Supplement on Rumen Metabolite Alterations Under Heat Stress Conditions

A total of 1,169 peaks were detected with GC–TOF–MS, and 1,150 of metabolites were further identified across all samples by retaining the treatments with null value ≤ 50%. All identified metabolites are provided in Supplementary Table 2. The metabolites mainly clustered into organic acids, fatty acids, carbohydrates, amino acids, purines, and biogenic amines.

**Figure 3:**
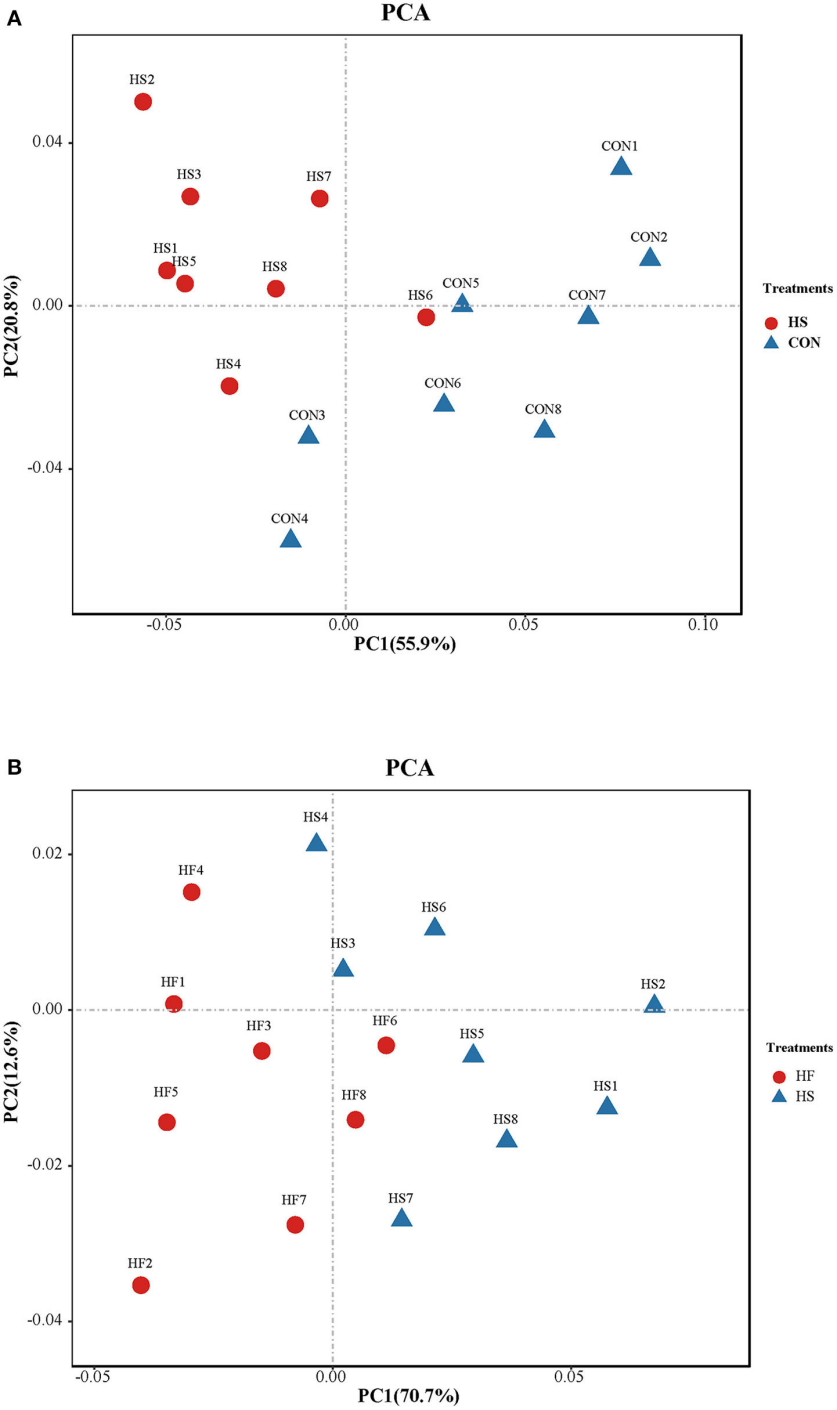
Principal components analysis (PCA) on ruminal metabolite content between different treatments. **(A)** Principal components analysis (PCA) on ruminal metabolites content between CON and HS treatments. CO, control treatment; HS, heat stress treatment. **(B)** Principal components analysis (PCA) on ruminal metabolite structures between HS and HF treatments. HS, heat stress treatment; HF, honeycomb flavonoids supplement treatment.

Differential analysis on the integrity of metabolite alteration of honeycomb flavonoids supplement under heat stress treatment was first conducted through PCA. As shown in Figure 3A, PC1 and PC2 accounted for 55.9 and 20.8% of the total variation, respectively. Integrally speaking, samples of HS treatment could clearly separate from that of CON, except HS 6, which indicated that rumen metabolites showed a significant alteration under heat stress conditions compared with CON. In addition, metabolites also showed a significant difference after HF supplement compared with those under heat stress treatment as shown in Figure 3B. PC1 and PC2 accounted for 70.7 and 12.6% of the total variation, respectively. Samples of HF treatment could clearly separate from HS based on PC1 and PC2, except HF6.

Thereafter, significantly altered metabolites under heat-stressing treatment compared with control, and those after HF supplement compared with HS, were analyzed based on the statistical standard of VIP >1 and *p*-value <0.05. All the results are shown in Tables 8, 9, respectively. A total of 35 significant differential altered metabolites including 16 downregulated and 19 upregulated metabolites were detected based on the filtering standard. The downregulated metabolites mainly related with the carbohydrate metabolism such as FMNH and *cis*-acetylacrylate; amino acid metabolism including isoleucyl-asparagine, dimethyl-l-arginine, and serinyl-threonine; and certain organic acids. Besides, most upregulated metabolites related with nucleotide metabolism, such as hypoxanthine, pyrimidin-2-one-ribonucleoside, 2-phenylacetamide, xanthine, thymidine, and a number of bioamines.

**Table 8:** Ruminal metabolite alterations under heat stress conditions.

	**Name**	**HS mean**	**CON mean**	**Fold change**	***P*-value**	**VIP**
Downregulation	Phenyl-1-thio-beta-D-galactopyranoside	3.99E-06	1.70E-05	0.23	0.001	2.041
	Zidovudine	5.84E-06	2.48E-05	0.38	0.001	1.795
	Dimethyl-L-arginine	3.37E-06	8.98E-06	0.43	0.001	1.751
	Pyridoxine	4.30E-06	1.00E-05	0.44	0.003	1.682
	Isoleucyl-asparagine	5.97E-04	3.17E-03	0.46	0.008	1.637
	Herculin	2.41E-06	1.16E-05	0.11	0.008	1.745
	Ferulic acid	4.06E-05	1.24E-04	0.33	0.010	1.563
	Methadone	1.94E-06	9.80E-06	0.19	0.019	1.622
	Hexanediol 1,6-bisphosphate	2.24E-05	5.64E-05	0.21	0.021	1.343
	Serinyl-threonine	2.59E-06	6.49E-06	0.33	0.024	1.497
	2,5-Dihydroxybenzoic acid	8.11E-03	4.49E-02	0.31	0.034	1.500
	Hippuric acid	1.26E-05	6.68E-05	0.18	0.034	1.229
	4-Acetamidobutanoic acid	2.91E-06	1.22E-05	0.24	0.038	1.245
	*cis*-Acetylacrylate	3.32E-05	7.50E-05	0.42	0.040	1.235
	FMNH	1.56E-03	3.43E-03	0.28	0.040	1.362
	3,4-Dihydroxybenzoic acid	1.88E-06	7.36E-06	0.29	0.042	1.405
Upregulation	7,8-Dihydroneopterin	1.59E-05	4.02E-06	3.94	0.000	2.087
	Androsterone sulfate	3.93E-05	1.23E-05	3.18	0.000	2.052
	2′-Deoxyuridine	5.54E-04	1.56E-04	3.55	0.000	1.913
	Dyspropterin	2.93E-04	3.64E-05	8.07	0.000	2.056
	Hypoxanthine	1.73E-04	7.91E-05	2.19	0.001	1.906
	Pyrimidin-2-one-ribonucleoside	9.35E-04	2.10E-04	4.45	0.001	1.822
	L-Phenylalanine	1.61E-03	7.89E-04	2.04	0.001	1.897
	1-Oleoyl-lysophosphatidic acid	2.88E-04	1.18E-04	2.44	0.001	1.919
	11-Keto-beta-boswellic acid	5.41E-05	1.45E-05	3.73	0.002	1.810
	L-Tryptophan	4.72E-04	2.58E-04	1.83	0.002	1.848
	2-Phenylacetamide	3.02E-04	1.16E-04	2.61	0.002	1.957
	L-Tyrosine	1.21E-03	4.50E-04	2.68	0.002	1.948
	Ribothymidine	1.71E-04	7.54E-05	2.28	0.004	1.597
	Tetradecanoylcarnitine	7.81E-05	1.21E-05	6.44	0.004	1.893
	Xanthine	4.47E-03	1.87E-03	2.39	0.004	1.828
	Thymidine	9.74E-03	4.16E-03	2.34	0.005	1.563
	4-Deoxypyridoxine 5′-phosphate	7.21E-05	2.61E-05	2.76	0.006	1.847
	3-(indol-3-yl)pyruvic acid	1.46E-05	7.25E-06	2.01	0.006	1.631
	5-Aminoimidazole ribonucleotide	6.22E-05	2.39E-05	2.61	0.009	1.659

*CON, control treatment; HS, heat stress treatment; FC, fold change; VIP, variable importance in the projection*.

**Table 9:** Ruminal metabolite alterations to the honeycomb flavonoids supplement under heat stress conditions.

	**Name**	**HS**	**HF**	**FC**	***P*-value**	**VIP**
Upregulation	PG [16:0/18:1(11Z)]	2.22E-06	1.03E-05	0.22	0.010	1.300
	PI [16:0/18:1(9Z)]	2.29E-03	3.90E-03	0.59	0.029	1.307
	PS (17:0/16:0)	1.28E-05	2.34E-05	0.55	0.043	1.153
	Daidzein	5.22E-06	1.25E-05	0.42	0.049	1.196
	2-Methylisoborneol	1.46E-04	2.49E-04	0.58	0.052	1.186
	PE (14:0/14:0)	1.47E-05	2.99E-05	0.49	0.059	2.687
	Serinyl-threonine	5.67E-05	1.21E-04	0.47	0.070	1.258
	Methadone	1.56E-03	3.56E-03	0.44	0.042	1.828
	Herculin	9.09E-05	2.15E-04	0.42	0.035	1.227
	9(S)-HOTrE	1.30E-05	3.15E-05	0.41	0.048	1.467
Downregulation	Prolyl-asparagine	6.98E-03	3.26E-03	2.15	0.030	1.979
	Dyspropterin	1.31E-04	3.53E-05	3.71	0.036	1.669
	N-Acetyl-D-muramoate	1.58E-04	7.32E-05	2.16	0.039	1.828
	N-Acetylglucosaminylasparagine	2.55E-05	8.60E-06	2.97	0.040	3.013
	Acyclovir	1.98E-05	8.85E-06	2.24	0.041	1.131
	Linoleoyl ethanolamide	1.73E-04	5.99E-05	2.89	0.041	2.153
	Rhapontigenin	2.49E-05	6.82E-06	3.65	0.050	1.025
	7,8-Dihydroneopterin	9.30E-06	3.16E-06	2.94	0.044	1.290
	Glycerophosphocholine	4.82E-05	1.35E-05	3.57	0.031	1.900
	Tetradecanoylcarnitine	3.55E-05	1.00E-05	3.54	0.024	1.874

*CON, control treatment; HS, heat stress treatment; FC, fold change; VIP, variable importance in the projection; PG, phosphatidylglycerol; PI, phosphatidylinositol; PS, phosphatidylserine; PE, phosphatidylethanolamine*.

The investigation on rumen metabolites changes after HF supplements were subsequently provided. As shown in Table 8, a total of 20 significant differential altered metabolites, including 11 upregulated metabolites and nine downregulated metabolites, were identified. The mainly upregulated metabolites are clustered into lipid metabolism, such as phosphatidylglycerol (PG), phosphatidylinositol (PI), phosphatidylserine (PS), and phosphatidylethanolamine (PE). Besides, the downregulated metabolites were mostly bioamines, which formed from protein degradation, and might insult ruminal epithelium when excessively accumulated.

**Figure 4:**
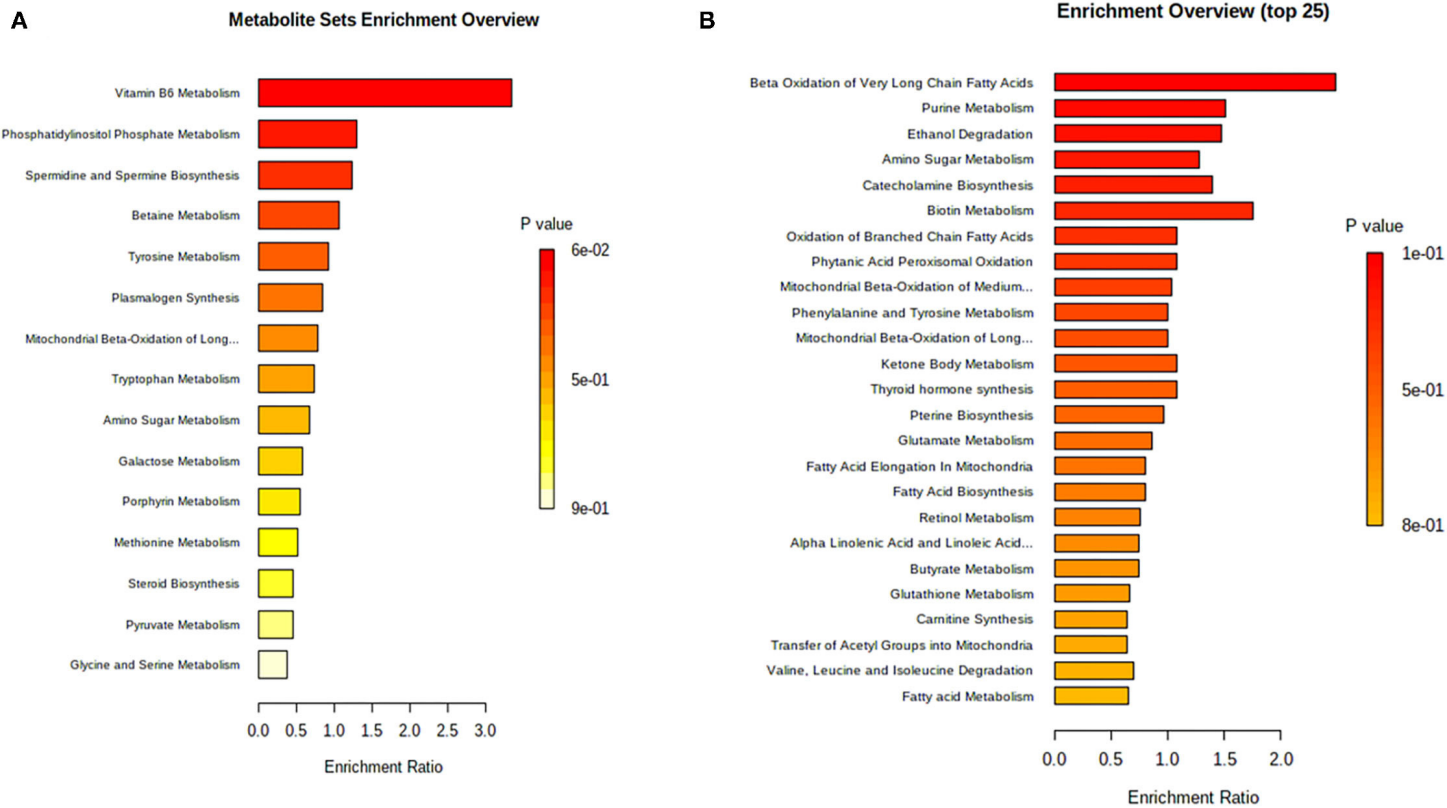
Functional enrichment analysis on differential ruminal metabolites. **(A)** Functional enrichment analysis on differential ruminal metabolites between CON and HS treatments. CON, control treatment; HS, heat stress treatment; *p* < 0.05 was considered as significant difference. **(B)** Functional enrichment analysis on differential ruminal metabolites after honeycomb flavonoids supplement under heat stress conditions. CON, control treatment; HS, heat stress treatment; *p* < 0.05 was considered as significant difference.

Finally, the functional enrichment analysis was applied based on all differential metabolites to determine the mainly altered functions under heat stress conditions and after honeycomb flavonoids supplement. All enrichment results are shown in Figure 4.

Figure 4A shows the critical altered functions enriched between heat stress and control treatments. Vitamin B_6_ metabolism, phosphatidylinositol phosphate metabolism, and spermidine biosynthesis accounted for the three most enriched functions, which might indicate the suppression of both carbohydrate metabolism and regular protein metabolism under heat stress conditions. Furthermore, functional investigation of honeycomb flavonoids supplement on ruminal metabolism was applied and shown in Figure 4B. The energy metabolism–related functions such as beta-oxidation of fatty acids, and the bacterial proliferation–related functions such as purine metabolism presented a major difference after honeycomb flavonoids supplementation. Besides, amino acids and their derivate-related function also showed a great promotion after HF treatment.

## Discussion

Heat stress is seriously detrimental to dairy production and economic benefits, especially in South China. When ambient temperature and humidity do not decrease enough, dairy cows are unable to lose heat and further cause them to be in a constant state of heat stress. Cows supplemented with Chinese medicines, which contain abundant flavonoids, showed better immune condition and alleviation effects under the heat stress environment (21). Therefore, in our present study, we purified flavonoids from honeycomb and supplemented it into dairy cows to determine both the nutrient digestibility and milk productivity responses under heat stress environments.

### Effects of Honeycomb Flavonoids Supplement on Rumen Microbial Homeostasis and Nutrient Degradability

Energy, which is mainly derived from nutrient ingestion, restricted neither the growth nor the production performances of dairy cows. Absence of energy provision under heat stress conditions directly suppressed rumen microbial proliferation and degradability, and thereafter disrupted ruminal homeostasis.

Indeed, ruminal homeostasis provided an ideal environment for nutrient digestion, transportation, energy generation, and the microbial proliferation (22, 23). Hence, ruminal microbiota generally showed adaptive responses to heat stress. Relative abundances of fiber-degrading bacteria including *Fibrobacteres* and *Bacteroidetes* considerably decreased because of the high energy dissipation and exothermic effects during the fiber degradation process, while starch-utilizing bacteria such as *Firmicutes* and *Streptococcus* showed more degradable activities (24, 25). Besides, heat-stressed cows also had a significantly higher relative abundance of lactate-producing bacteria (e.g., *Streptococcus*), which utilize soluble carbohydrate as an energy source (26). All these adaptive changes were also observed in the HS treatment compared with CON, which affirmed the occurrence of heat stress. Microbial changes consequentially resulted in lower VFA production and energy provision. The relative abundance of the acetate-producing bacterium *Acetobacter* decreased with heat stress treatment (16). Such a finding was also observed in the present study and may give proof of the decrease of acetate content.

All the aforementioned drawbacks may be conversed after flavonoids supplement for the regulatory effects on microbial proliferation. Generally, flavonoids are presented in their glycoside forms, which were further deglycosylated by β-glucosidases secreted by microbiota and resulted in the increase of aglycons (27). The aglycons were further utilized by ruminal bacteria to enhance proliferation of bacteria. Interestingly, HF supplement in the present study significantly increases both cellulolytic and amylolytic bacteria. These alterations help recover microbial construction and further energy metabolism systems, which were reflected in the more abundant content of total VFAs and acetate. These changes might be attributed to the increase of α-diversity that utilized more carbohydrates into VFAs and the increase of rumen cellulolytic bacteria. For the ruminal acetate, it was primarily derived from cellulose degradation (28).

Besides, as shown by metabolomics result, beta oxidation of fatty acids, which could further generate acetyl-CoA for energy provision (29), was significantly enhanced after HF supplement. Fatty acids possessed superior energy to carbohydrates and might supply more energy for rumen microbial proliferation; thus, the re-establishment of micro-ecosystem could be recovered.

In addition, proliferated microbiota enhanced ruminal protein utilization. Rumen cellulolytic bacteria have been proven to use ammonia as their sole nitrogen source because the incorporation of preformed amino acids is minimal (30). The proliferated cellulolytic bacteria consumed more ammonia, which causatively induced the reduction of bioamines after HF supplement, as aforementioned in the metabolomics results. Moreover, the promoted ammonia utilization catalyzed protein degradation; therefore, nitrogen utilization was enhanced.

### Effects of Honeycomb Flavonoids Supplement on Milk Production and Milk Quality

Milk production is superior to any other parameters for the dairy cattle industry because of both economic and effective factors. DMI and gastrointestinal absorptivity contributed the most on milk production. In our research, honeycomb flavonoids promoted milk production; reasons could be attributed to enhancing nutrient absorptivity.

Nutrient absorptivity is mainly affected by epithelial transporting capacity. The epithelial barrier and transporting functions were disrupted under heat-stressed conditions by severe inflammatory responses because of the lipopolysaccharide release caused by the death of bacteria (31). Flavonoids may benefit from the epithelial integrity and transporting efficiency because of their mighty anti-inflammatory capacities, which therefore enhanced nutrient absorptivity (32). In addition, bile acid (BA) metabolism might be another causative factor. In recent research, the BA signaling pathway was proved to be critical in both lipid and carbohydrate metabolism, also in maintaining esoteric glucose and cholesterol homeostasis as well as immune states (33). BA is also a crucial secondary metabolite that is utilized by gut microbiota and communicated with epithelial cells. Intriguingly, flavonoids could be responsible for increased BA production (6), and further help promote lipid and carbohydrate metabolism to provide more energy for milk production.

Milk fat content increased after HF supplement. Milk fat is a necessary parameter of milk quality and beneficial to human health, and is mostly formed from acetate generated by ruminal fermentation (28). Based on the previous finding, acetate is mainly generated by cellulose degradation (16), and the increasing cellulose-degrading bacteria, especially the mighty acetate-generating bacteria *Acetitomaculum*, significantly increased after HF supplement, which finally resulted in the increase in acetate content and further milk fat content.

Moreover, improved ruminal lipid metabolism, as showed in our metabolomics result, may be another causative factor for the increase of milk fat content. When dietary lipids enter the rumen, the initial step in lipid metabolism is the hydrolysis of the ester linkages found in TG, phospholipids, and glycolipids, and this is primarily carried out by hydrolases produced by rumen bacteria (34). The hydrolyzed lipid was further transported into mammary gland and synthesized milk fat as substrates. Therefore, milk fat content greatly increased after honeycomb flavonoids supplement.

## Conclusion

In summary, production performances were seriously suppressed under heat stress conditions and caused huge economic loss. Honeycomb flavonoids supplement help proliferate microbial abundances and fiber-degrading bacteria, which further promoted nutrient degradability and energy metabolism, and ultimately enhanced the production performances of dairy cows. These findings in the present study might provide a proper selection for dairy production under heat stress conditions.

## Data Availability Statement

The data presented in the study are deposited in the NCBI Sequence Read Archive (SRA, http://www.ncbi.nlm.nih.gov/Traces/sra/), accession number PRJNA753017.

## Ethics Statement

The animal study was reviewed and approved by Animal care and procedures followed the Chinese Guidelines for Animal Welfare, which was approved by the Animal Care and Use Committee of Jiangxi Agricultural University, with the approval number JXAULL-20210619.

## Author Contributions

EL and HW designed the study. EL, MS, CH, KM, and QL conducted the experiment. JZ, DW, SW, CZ, WL, and SG participated in parameter measurement and data analysis. FX wrote the article and contributed to English editing. All authors contributed to the article and approved the submitted version.

## Funding

The study was funded by the Science and Technology Planning Project of Jiangxi Educational Department (GJJ200414).

## Conflict of Interest

The authors declare that the research was conducted in the absence of any commercial or financial relationships that could be construed as a potential conflict of interest.

## Publisher's Note

All claims expressed in this article are solely those of the authors and do not necessarily represent those of their affiliated organizations, or those of the publisher, the editors and the reviewers. Any product that may be evaluated in this article, or claim that may be made by its manufacturer, is not guaranteed or endorsed by the publisher.

## Supplementary Material

Supplementary Table 1 Taxonomy results of ruminal bacteria community.

Supplementary Table 2 Identification results of ruminal metabolite responses to honeycomb flavonoids supplement.
